# Securing cloud data using secret key 4 optimization algorithm (SK4OA) with a non-linearity run time trend

**DOI:** 10.1371/journal.pone.0301760

**Published:** 2024-04-16

**Authors:** Twum Frimpong, James Benjamin Hayfron Acquah, Yaw Marfo Missah, John Kwao Dawson, Ben Beklisi Kwame Ayawli, Philemon Baah, Samuel Akyeramfo Sam

**Affiliations:** 1 Department of Computer Science, Kwame Nkrumah University of Science and Technology, Kumasi, Ghana; 2 Department of Computer Science, Sunyani Technical University, Sunyani, Ghana; University of the West of Scotland, UNITED KINGDOM

## Abstract

Cloud computing alludes to the on-demand availability of personal computer framework resources, primarily information storage and processing power, without the customer’s direct personal involvement. Cloud computing has developed dramatically among many organizations due to its benefits such as cost savings, resource pooling, broad network access, and ease of management; nonetheless, security has been a major concern. Researchers have proposed several cryptographic methods to offer cloud data security; however, their execution times are linear and longer. A Security Key 4 Optimization Algorithm (SK4OA) with a non-linear run time is proposed in this paper. The secret key of SK4OA determines the run time rather than the size of the data as such is able to transmit large volumes of data with minimal bandwidth and able to resist security attacks like brute force since its execution timings are unpredictable. A data set from Kaggle was used to determine the algorithm’s mean and standard deviation after thirty (30) times of execution. Data sizes of 3KB, 5KB, 8KB, 12KB, and 16 KB were used in this study. There was an empirical analysis done against RC4, Salsa20, and Chacha20 based on encryption time, decryption time, throughput and memory utilization. The analysis showed that SK4OA generated lowest mean non-linear run time of 5.545±2.785 when 16KB of data was executed. Additionally, SK4OA’s standard deviation was greater, indicating that the observed data varied far from the mean. However, RC4, Salsa20, and Chacha20 showed smaller standard deviations making them more clustered around the mean resulting in predictable run times.

## 1. Introduction

**Fig 1:**
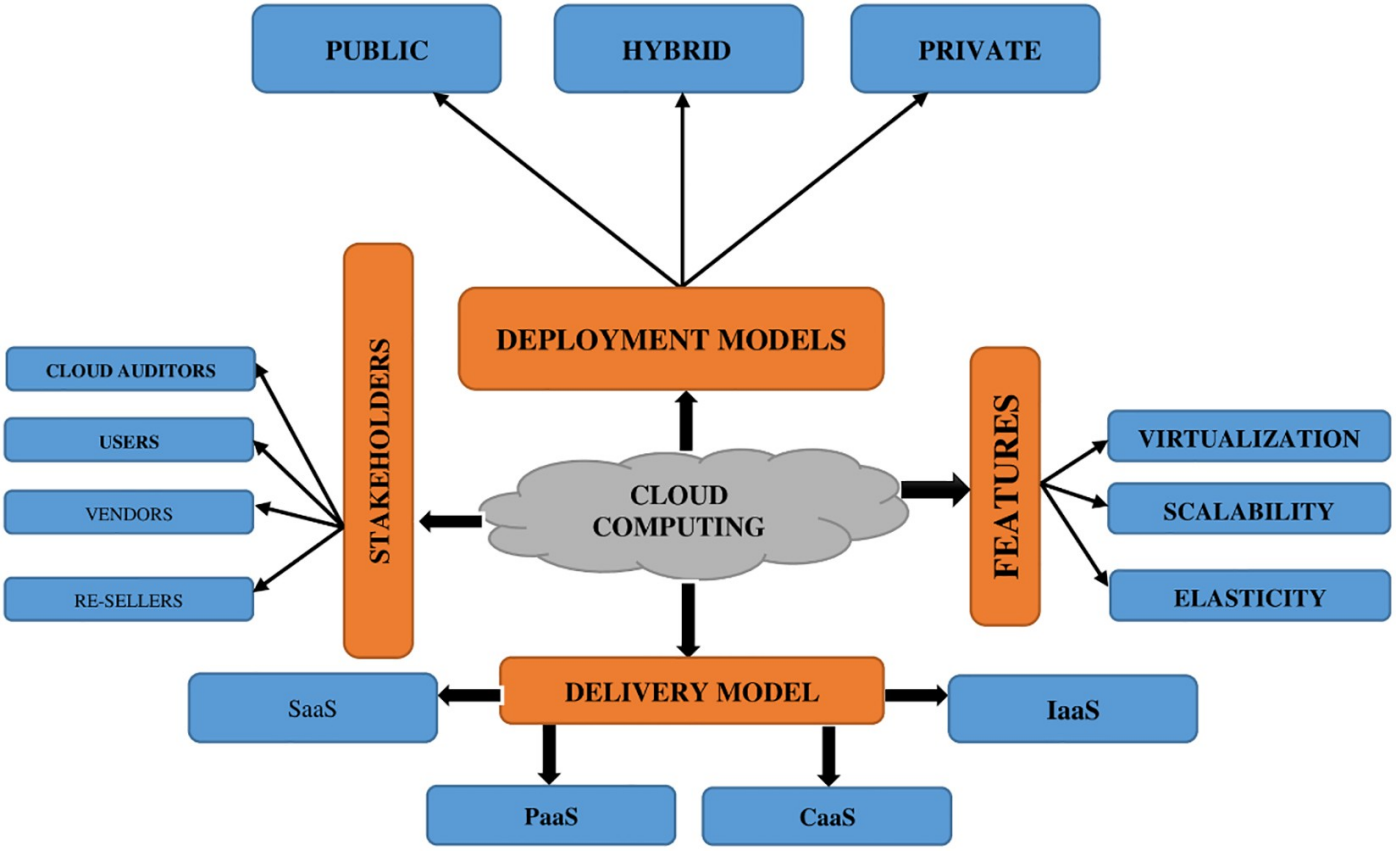
Cloud computing architecture and features.

Cloud computing is an environment in which all data is stored, processed, and produced via a network of distant computers distributed throughout the Internet. It offers a practical solution for data access and storage from anywhere via Internet-connected devices [1]. Many firms are switching from traditional data storage to cloud storage since it allows for quick access to information from anywhere. Cloud computing has been critical to driving digital transformation and enabling cutting-edge technologies such as Artificial Intelligence (AI) and Machine Learning (ML) [2]. Because of the sharp rise in users, cloud computing has seen a significant increase in communication, necessitating a more effective way of data transfer and securing data. Cloud computing users have expanded owing to its numerous benefits, such as scalability, collaboration, reduced expenses, and flexibility; as seen in the conceptual and cloud computing features in Fig 1. These advantages are offered through cloud deployment techniques such as public cloud, hybrid cloud, and private clouds, delivered through delivery models such as Software-as-a-Service (SaaS), Platform-as-a-Service (PaaS), Infrastructure-as-a-Service (IaaS), and Container-as-a-Service (CaaS).

Platforms for cloud computing include a large concentration of users and information resources, making them attractive targets for hackers. For instance, the cloud service provider Blackbaud fell victim to a Ransom ware attack in 2020 which prohibited users from accessing data and servers. The hackers hacked into their networks and tried to install Ransom ware. This attack resulted in significant financial losses and user privacy leaks [2, 3].

Cloud computing security risks are becoming increasingly prevalent. The security of cloud computing is a problem that is currently generating research topics. Cloud computing security considerations include end-user data security, network traffic security, file system security, and host system security. The employment of algorithmic techniques in process and data systems is becoming increasingly visible as worries about cloud services and information security grow [4]. Encryption is one of the most effective techniques for data security and privacy preservation. Encrypted data is safeguarded because it is altered and sent through cryptography, rendering it unreadable to unauthorized users [5].

The use of encryption algorithms help maintain cloud data access control, cloud data trust management, verified computing, cloud data authorization, authentication, and safe data storage [6, 7]. However, because run times is dependent on data size (O (N)), these cryptographic algorithms add overhead expenses to cloud infrastructure [8]. This has an influence on the Quality of Service, including performance and security [9]. Also, this causes wear and tear on cloud equipment as well as the demand for huge data bandwidth to transfer data, which adds on to the costs of cloud providers and cloud clients [8].

### 1.1 Identified problem

A developing and innovative method of providing offshore computer and storage services, which has recently become more risky in terms of security is cloud computing. When a third party controls and manages data and assets of an organization, a number of risks including those related to confidentiality, privacy, data leakage, data theft, reliability, capacity, and performance evaluation, are present. Researchers have suggested cryptographic algorithms as suitable tools for assuring the security of subscribers’ data on the cloud, as a result of these security issues. Although several cryptographic techniques have been proposed by professionals, security still prevents cloud computing from being widely used [10, 11]. Again, the relationship between run time and data size for these cryptographic schemes suggests that run time increases linearly with an increasing data size (O (N)) [12–15]. In addition, predictable run times allow hackers to attack vulnerabilities based on execution time hypothesis.

### 1.2 Novelty of proposed algorithm

Research has demonstrated that data size affects run time [14, 39] proportionally.

In light of these considerations, this paper presents a lightweight, non-linear(*F* − *kv*^2^) stream cipher with shorter run times dubbed Secret Key 4 Optimization Algorithm (SK4OA). This cryptographic scheme is an integration of Cousin Primes, Pseudo Random Number Generator, Sliding Window Algorithm, Greatest Common Divisor and the XOR circuit gate. The integration helps to improve the security of cloud infrastructure and safeguard cloud data. Again, the cryptographic scheme has the potential to utilize less bandwidth to transfer large amounts of data since the run time is determined by the secret key rather than the data size. Furthermore, because the execution times of this cryptographic technique is unpredictable, it will be able to withstand security attacks such as brute force.

Therefore, the contribution of this research is as follows;

- The existing algorithms are linear causing the run time to increase linearly as the data size increases [12–15]. The proposed Secret Key 4 Optimization Algorithm (SK4OA) is non-linear which makes the run time independent of data size leading to less bandwidth use in data transfer.
- The existing algorithms give room for predictable run times that allow hackers to attack vulnerabilities based on execution time hypothesis. However, the proposed SK4OA algorithm being nonlinear prevents such predictions and makes it resistant to attack.

## 2. Literature review

Data sharing in the cloud is a well-known way to give people and businesses scalable, limitless storage and computing capabilities. The use of the cloud, however, also raises a number of security and privacy issues, including data integrity, confidentiality, dependability, fault tolerance, and other issues. The most secure method of thwarting these security concerns is the employment of cryptographic techniques.

Sajay, Babu, and Vijayalakshmi proposed a hybrid approach to improving cloud data security that uses an encryption method. To enhance cloud security, their work coupled homographic and blowfish encryption [16]. Encryption approaches were generally used to safeguard or store large volumes of data on the cloud. Their approach was unique, yet the execution times were long and linear.

An effective real-time service-centric feature sensitivity analysis (RSFSA) technique was put up by Siva Kumar et al. [17], to achieve cloud data security. The RSFSA model examined how sensitively certain characteristics were used by various services at various levels. The method computed the FLAG value for the user in accordance with the provided profile by checking the set of features being accessed at each level and the number of features to which the user has access permissions. The user had either been given access to the service or not, depending on FLAG’s value. Although their method had significantly reduced run times, the trend of the run time was linear, making it predictable. The authors [18] integrated a nature-inspired optimization, such as a moth search algorithm (MSA) with ECC, to choose the right and ideal value of the elliptic curve in order to provide more secure data encryption. The DNA encoding and ECC means of encryption were combined in the suggested scheme. With less computing resources, the DNA-encoded ECC technique offered multi-level security. Anuj Kumar’s study in 2021 also suggested combining a DNA-based algorithm and the AES Algorithm. Data encryption and decryption were performed using DNA cryptography technology and the AES strategy [19]. The proposed algorithm attained cloud security but the trend of the execution times was linear. Deoxyribonucleic acid (DNA)-based cryptographic scheme was also suggested by Joseph and Mohan [20], as a way to improve data security when sharing data over the internet. The Grey Wolf Optimization (GWO) Algorithm was used in this process to implement an optimized encryption model in order to produce the best encrypted data while sharing. Kumar’s and Joseph’s study were very novel, however their computational times was linear.

Vidhya and Mohan Kumar [21] suggested using a Fusion-based Advanced Encryption Algorithm (FAEA) as a cost-effective, practical security solution for using Big Data in the cloud. The FAEA technique outperforms the existing Hadoop Distributed File System (HDFS) and Map Reduce Encryption Scheme (MRE) by 98%, according to an examination of its efficiency, scalability, and security. The results of their cloud deployment revealed that run times were linear (O(N)), with run times dependent on data size. Adee and Mouratidis’ [22], paper suggested combining the Least Significant Bit steganography with the RSA cryptographic system, Advanced Encryption Standard, and identity-based encryption techniques. The four phases were data sharing, data backup and recovery, steganography, and data encryption to safeguard and secure cloud data. The proposed scheme was novel however, the run time was linear.

In the study of Kaur et al. [6], a triple encryption strategy to attain cloud data security was proposed. The scheme was designed to offer complete data protection across the whole data life cycle, including data in storage and transit. They created a hash value using the SHA256, AES, and XOR processes to protect the privacy of cloud data. Despite the potentials of their scheme, they had lengthier and linear run times. In the same year, Guo et al. [23], proposed a cryptographic scheme using the Blockchain-aided ABE with escrow-free (BC-ABE-EF) system, to achieve cloud data security. Their strategy primarily overcame the key escrow issue by substituting a consortium Blockchain for the established key authority. A secure key-issuing protocol was used to produce the keys between the Blockchain and the data user. The Blockchain could not access the user’s whole key on its own but used the decryption cloud server to plan pre-decryption tasks as well. Additionally, their system, added a group manager to update the group keys of users which could not be retrieved and created re-encryption keys. Their investigation led to the conclusion that the relationship between data size and run-time cost was directly proportional. A hybrid technique that combined RSA with the Gaussian Interpolation Formula was suggested by the authors in the same year, 2022, to ensure the security of cloud data [24]. Although the algorithm’s runtime was shorter, it was predictable because of its linear run time trend.

To encrypt data to attain cloud security, Kousalya and Baik [25] recommended utilizing Improved RSA-based role-based access control (RBAC) with extended access connectivity markup language (XACML). This method enabled the connected computer to store data using cryptographic principles and data made accessible through a simple admission management system. Sensitive data was protected globally, thus a technique of encryption was used that combined a traditional homogeneous encryption approach with an unstable information dissemination mechanism. Their proposed scheme was able to achieve the targeted objective but the run time was linear. The use of a Cloud Secure Storage Mechanism (CSSM) to ensure cloud security was also suggested by Ramachandran et al. [26]. Data invasions at the storage layer were prevented via CSSM’s provision of encrypted, chucked, and scattered storage, which combined data dispersion with distributed storage to attain cloud data security. In order to prevent any illegal access to cryptographic materials, CSSM also utilized a system of management levels, user passwords, and secret sharing method. The combination of mCrypton with salsa20 was suggested by Hameed and Hoomod [27], as a way to safeguard data stored in the cloud. Their algorithm was a simple one that could be used in embedded systems. However, because run time is dependent on data amount, the linear run time might potentially harm the performance of these systems when large sizes of data are to be transmitted. A hybrid cryptosystem based on the fusion of the RSA and DNA algorithms was suggested by Bhati et al. in 2023 [28]. They combined the benefits of symmetric-key (private-key) and asymmetric-key (public-key) cryptosystems in their technique. Despite being a revolutionary algorithm, their execution time depended on the amount of data executed. In the study of [29], the authors introduced the Soldier Ant Algorithm (SAA), which is a hybrid algorithm. The Diffie-Hellman algorithm and the Newton Forward and Backward Interpolation (Delta Encoding) method were integrated to improve cloud data security. Although their approach could withstand man-in-the-middle attack, the runtime was based on the size of the data to be executed.

Kumar et al. [30] proposed a deoxyribonucleic acid (DNA) computing to achieve data security. A 512-bit secret key was generated and used for data encoding by the owner of the data, which was later outsourced to the cloud. Although the algorithm performance is promising, the run time trend is linear.

According to the linked studies reviewed, all of the proposed solutions by various researchers were capable of ensuring cloud data security. However, the linearity of the run times (O (N)) is a drawback shared by all of these cryptographic techniques. Data size and run times had a clear correlation that lead to cloud infrastructure tear and wear. Once more, predictable run times provide hackers the chance to exploit vulnerabilities based on execution time hypotheses. Table 1 provides an overview of the relevant works, including the Author(s), the year of publication, the security issue to be resolved, the used cryptographic system, and the run time trend.

**Table 1:** A comparison of cryptographic algorithms for cloud data security.

Author (s)	Year of Publication	Security Challenge	Scheme	Run Time Trend
Sajay, Babu & Vijayalakshmi [16]	2019	Cloud Data Security	Homographic encryption and blowfish encryption	Linear
Siva Kumar et al. [17]	2021	Cloud Data Security	Real-Time Service-Centric Feature Sensitivity Analysis	Linear Lower Run time
Kumar & Kumar Bhatt [18]	2020	Cloud Data Security	DNA encoding with ECC encryption algorithm	Linear
Anuj Kumar [19]	2021	Cloud Data Security	DNA-based algorithm and the AES Algorithm.	Linear
Joseph & Mohan [20]	2022	Cloud Data Security	Deoxyribonucleic acid (DNA)-based cryptographic	Linear
Vidhya &Mohan Kumar [21]	2022	Cloud Data Security	Fusion-based Advanced Encryption Algorithm (FAEA)	Linear
Adee and Mouratidis [22]	2022	Cloud Data Security	RSA, AES and Steganography	Linear
Kaur et al. [6],	2023	Cloud Data Security	Triple Encryption Strategy	Linear
Guo et al. [23],	2023	Cloud Data Security	Blockchain-aided ABE with escrow-free (BC-ABE-EF)	Linear
Dawson et al. [24]	2022	Cloud Data Security	RSA and Gaussian Interpolation Formula	Linear
Kousalya & Baik [25]	2023	Cloud Data Security	Improved RSA-based role-based access control (RBAC) with extended access connectivity markup language (XACML).	Linear
Ramachandran et al. [26]	2023	Cloud Data Security	Cloud Secure Storage Mechanism	Linear
Hameed & Hoomod [27]	2023	Cloud Data Security	Integration of mCrypton and salsa20	Linear
Bhati et al. [28]	2023	Cloud Data Security	Hybrid RSAand DNA	Linear
Dawson et al. [29]	2023	Cloud Data Security	Diffie-Hellman Algorithm and Newton Forward and Backward Interpolation	Linear
Kumar et al. [30]	2023	Cloud Data Security	Deoxyribonucleic acid (DNA) technique	Linear

## 3. Methodology

In this paper a Security Key 4 Optimization Algorithm (SK4OA) is proposed to ascertain the security of cloud data. Security Key 4 Optimization Algorithm (SK4OA) is able to give accurate results irrespective of the noise *F*^*N*^(*X*) present in the chosen dataset as compared to other algorithms that output inaccuracies in many real-world contexts owing to noise [31]. SK4 Optimization Algorithm (SK4OA) is an integration of Cousin Prime [32], Pseudo Random Number Generator (PRNG), Fixed Sliding Window Algorithm, Great Common Divisor and XOR circuit gate.

**Fig 2:**
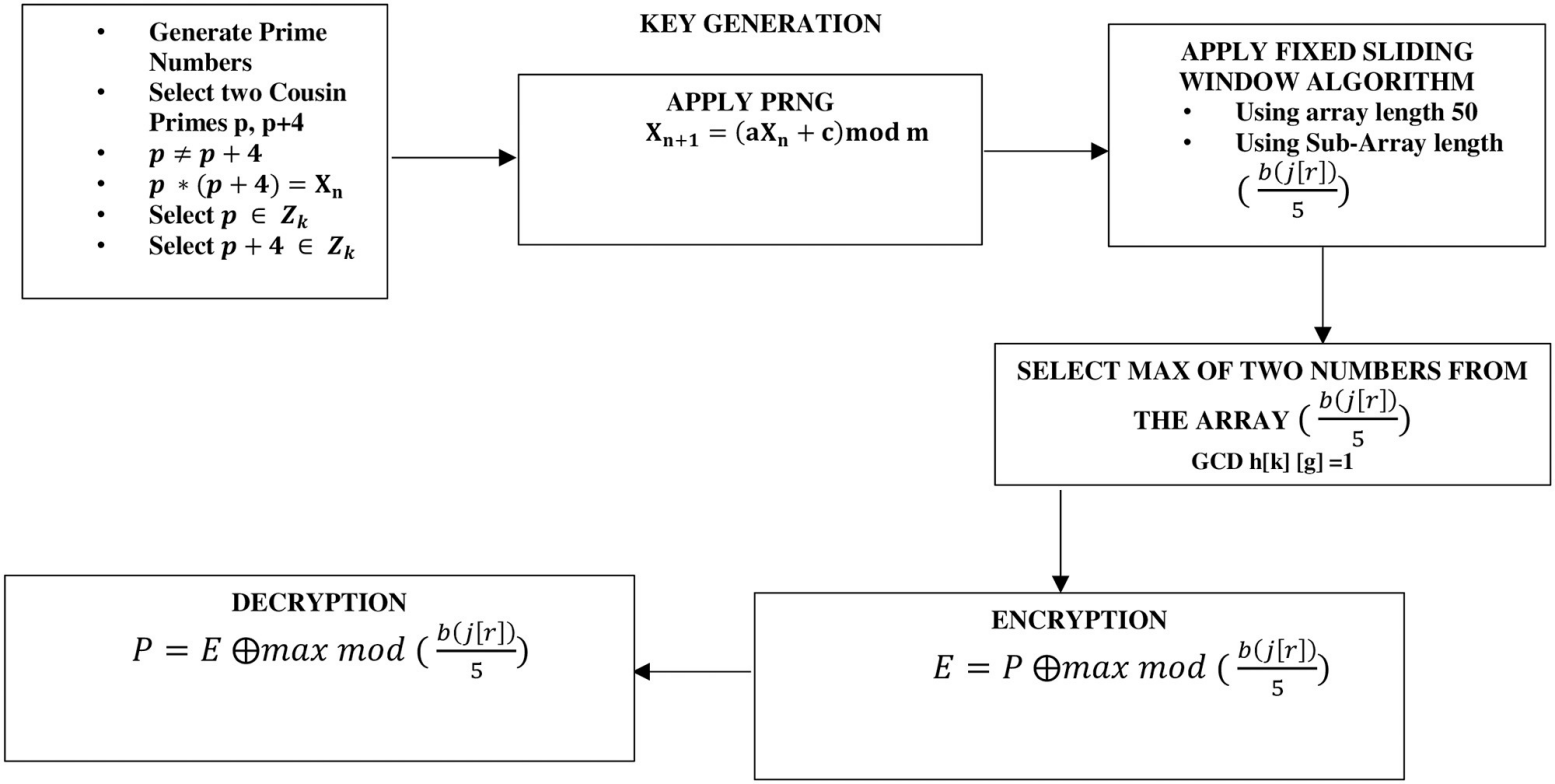
Work flow diagram for proposed algorithm SK4OA.

SK4OA has four levels of key generation and an encryption and decryption levels. The four levels for the generation of the secret key aims at strengthening the security of the proposed algorithm. In this algorithm, two cousin primes are selected and their products computed. The resultant is used as a seed value for the PRNG to generate 100,000 numbers. From the 100,000 numbers generated, fifty (50) numbers are randomly selected. The Sliding Window Algorithm is used to select ten (10) numbers from the randomly selected fifty (50) numbers using a sub-array of $\left(\;\frac{b\left(j\left[r\right]\right)}{5}\right)$. The maximum of two numbers whose Greatest Common Divisor (GCD) is one (1) is selected as a secret key for the encryption and decryption of the data applying a sub-array of $\left(\;\frac{b\left(j\left[r\right]\right)}{5}\right)$ as shown in Fig 2 and Algorithm 1.

**Algorithm 1**: SK4OA

 **Input**: P = *Plaintext*, *p = prime number*

**1**: Select (p, p+4) *// Cousin Prime*

**2**: *X*_*n*_ = *Product* (*p*, *p* + 4)

**3**: *X*_n+1_ = (*aX*_*n*_ + *C*)% *m*     *// Compute PRNG*

**4**: While *i* ≤ 100000

  {

**5**:   *y* = *Rand* (*X*_*n*+1_)%*m*

**6**:   *i*++

**7**:  }

**8**: end while

**9**: *z* = *Rand* (*y*, 50)

**10: $k=\left(\;\frac{b\left(j\left[r\right]\right)}{5}\right)$**     // *(2)*.

**11**: if *GCD h*[*k*][*g*] = 1// *GCD = 1*

  {

  maxvalue = *max* (*h*[*k*][*g*])

  }

**12**: *E* = *P* ⨁*max* % (*b*(*j*[*r*])/5)     // *Encryption 6*

**13**: *P* = E ⨁*max* % (*b*(*j*[*r*])/5)     // *Decryption 7*

  **Output**: *Plaintext* (P)

### 3.1 Key generation

The Key Generation stage is made up of four stages. This helps to strengthen the security of SK4OA.

#### 3.1.1 Stage 1: Cousin prime generation

Cousin Prime are prime numbers that differ by four [26]. For instance let *n* ≥ 3 be a given integer. The proposition 1 is true to be considered a Cousin Prime.

*Proposition* 1:

(*n*, *n* +4) is considered pair of cousin primes when the tuple *C*^(*n*)^ contains neither 0 nor 2 [33].


$$P^{\left(n\right)}\left(k\right)\;\neq 0,2\;\forall k=1,\ldots .m$$

The products of any two generated Cousin Primes (*P*, *P* + 4) are used as the seed value for the Pseudo Random Number Generator (PRNG)

#### 3.1.2 Stage 2: Pseudo random number generator (PRNG)

Pseudo-Random Number Generator (PRNG) is a computational function that uses a deterministic procedure to produce a series of random integers [34]. Because most cryptographic protocols need the production and use of private values that must be kept hidden from outsiders, PRNG is employed in this cryptographic scheme to generate 100, 000 numbers. During the creation of the series of values between *R*_0_, *R*_1_, *R*_2_ as well as m- 0, m-1, the recursive relation of Eq 1 is used.

$$X_{n+1}=\left(aX_{n}+c\right)mod\;m$$ (1)

where *X*_*n*_ the product of the Cousin Primes, *m* − *moduls*, *a* − *multiplier*, *c* − *increment* fulfilling the conditions:

m,0<m−moduls

a,0<a<m−multiplier

c,0≤c<m−increment

$$X_{n,}-product\;of\;Cousin\;Prime$$

The Fixed Sliding Window Algorithm is then applied on a set of fifty (50) numbers selected randomly from the 100,000 numbers generated.

#### 3.1.3 Stage 3: Application of sliding window algorithm

A sliding window algorithm is applied when determining the outcomes for a variety of integers in an array. The goal of this is to minimize the complexity of time from (*O* (*t*^2^) to O (t) by combining several nested loops into a single loop. In this scheme a sub-array as indicated in Eq 2 is applied on the fifty (50) numbers to generate ten (10) numbers

$$\left(\;\frac{b\left(j\left[r\right]\right)}{5}\right)$$ (2)

$$b\left[j_{r}\right]=b_{s}+\left(5r\right)+\;b_{s}+\left(5r+1\right)+b_{s}+\left(5r+2\right)+b_{s}+\left(5r+3\right)+b_{s}+\left(5r+4\right),$$ (3)

where s = {0, 1, 2…49} representing the list of 50 randomly selected numbers

b = {0, 1, 2…9} indicating the list of the sum of the each five (5) numbers

r = {0, 1, 2…4} showing the list of the first five (5) numbers

This is expansion of Eq 2 is demonstrated in Eq 4 as;

$$b\left[j_{r}\right]=\left\{\begin{array}{c} b_{o}+b_{j+1}+b_{j+2}+b_{j+3}+b_{j+4}\\ b_{j+5}+b_{j+6}+b_{j+7}+b_{j+8}+b_{j+9}\\ b_{j+10}+b_{j+11}+b_{j+12}+b_{j+13}+b_{j+14}\\ b_{j+15}+b_{j+16}+b_{j+17}+b_{j+18}+b_{j+19}\\ b_{j+20}+b_{j+21}+b_{j+22}+b_{j+23}+b_{j+24}\\ b_{j+25}+b_{j+26}+b_{j+27}+b_{j+28}+b_{j+29}\\ b_{j+30}+b_{j+31}+b_{j+32}+b_{j+33}+b_{j+34}\\ b_{j+35}+b_{j+36}+b_{j+37}+b_{j+38}+b_{j+39}\\ b_{j+40}+b_{j+41}+b_{j+42}+b_{j+43}+b_{j+44}\\ b_{j+45}+b_{j+46}+b_{j+47}+b_{j+48}+b_{j+49} \end{array}\right.$$ (4)

#### 3.1.4 Stage 4: Conditioning using greatest common divisor (GCD)

Select the maximum of any two numbers from the ten arrays whose GCD is 1 using Eq 5.

by+cv=1, (5)

where b, y, c and v are integers.

*Proof*

If b,c∈ℤ

$$m=gcd\;\left(b,c\right)$$

$$∴\frac{f}{b}and\frac{f}{c}$$

$$\frac{f}{by+cv}where\;f/1$$

∴f=±1

Thus the greatest common divisor for *f* = 1

The maximum value selected from the application of the GCD is used as the secret key for the encryption and decryption process.

### 3.2 Encryption

At the encryption level, the modulus of the maximum value and the sub-array $\left(\;\frac{b\left(j\left[r\right]\right)}{5}\right)$ are computed, and then XORed with the ASCII values of the plaintext using Eq 6.

$$E=P⊕max\;mod\;\left(\;\frac{b\left(j\left[r\right]\right)}{5}\right)$$ (6)

Where E is the Ciphertext and P the plaintext

### 3.3 Decryption

The decryption formula is applied on the Ciphertext using Eq 7.


$$P=E⊕max\;mod\;\left(\;\frac{b\left(j\left[r\right]\right)}{5}\right)$$ (7)

The modulus of the maximum value and the sub-array $\left(\;\frac{b\left(j\left[r\right]\right)}{5}\right)$ is computed. The output binary values are XORed with the eight bit binary values for the ASCII of the Ciphertext to produce the plaintext.

### 3.4 Security analysis of SK4OA

We provide a quick analysis of our suggested algorithm’s impact on cloud data security in this part. Number randomization in SK4OA is the primary security defense against brute force attacks in cloud computing. By employing SK4OA, a hacker cannot access any message since each message’s execution time is randomly generated based on 100,000 numbers, of which 50,000 are randomly chosen using a sub-array of $\left(\;\frac{b\left(j\left[r\right]\right)}{5}\right)$ to produce 10 numbers. The secret key for the encryption and decryption of the massage is difficult, if not impossible, to construct. As a result, constraining the maximum and lowest numbers whose GCD is 1 helps to increase SK4OA security. The hacker must create a secret key by applying the sliding window technique in order to decipher the Ciphertext and carry out illegal data access. Since the secret key is produced at random, the hacker is unable to produce it. As a result, SK4OA is protected from brute force attacks.

Theorem 1: SK4OA is semantically protected against brute force attack

The hacker has to be able to calculate the product of any two randomly chosen cousin primes (*n*, *n* + 4) and the product used as seed for the PRNG equation *X*_*n*+1_ = (*aX*_*n*_ + *C*)*mod m*. This is based on the criteria *m*, 0 < *m*, 0 < *a* < *m*, 0 ≤ *c* < *m* in order to create 100,000 numbers. This is nearly difficult as, whenever two cousin primes are chosen, the seed value will continuously changing, increasing the computing time required for the seed value during the brute force process.

The sliding window algorithm makes it nearly impossible to generate the maximum and minimum numbers for the generation of the secret key for the encryption and decryption by using a sub-array of $\left(\;\frac{b\left(j\left[r\right]\right)}{5}\right)$ to compute (10) numbers from (50) randomly selected numbers from the (100,000) numbers. The comparison of the maximum and minimum values whose GCD is (1 raises the security of SK4OA by making it practically harder to use brute force to defeat the SKO4A method. Consequently, the suggested SK4OA is capable of withstanding a brute force attack.

## 4. Results

This section presents the datasets and experimental environment, and simulation results of the proposed SK4OA algorithm.

### 4.1 Datasets and experimental environment

This study’s dataset was derived from the Kaggle database [35]. The dataset is an English-to-French translation that incorporates text, numbers, and special characters. The dataset was used to evaluate the resilience of the algorithm in terms of runtime trend, memory utilization, and throughput. The proposed algorithm was evaluated using data sizes ranging from 3KB to 5KB, 8KB to 12KB, and 16 KB. The data was executed thirty (30) times using the proposed Secret Key 4 Optimization Algorithm (SK4OA) to assess the validity of runtime parameters, and their mean *μ* and standard (*σ*) deviation computed.

Our experiment was executed on an i7 Lenovo computer with a 2.10GHz CPU and 8GB of RAM. The programming language used for this experiment is the Hypertext Preprocessor (PHP) programming language. A message size of 3KB, 5KB, 8KB, 12KB, and 16KB was used to test the proposed scheme as used in the study of [36]. Tables 2 and 3 depict the mean (*μ*) and standard deviation (*σ*) of 30 different encryption and decryption times generated from SK4OA, the proposed cryptographic scheme in this study.

**Table 2:** Total encryption and mean and standard deviation (*μσ*) times for proposed SK4OA.

File Size (KB)	Total Encryption Time (ms)	Mean and Standard Deviation (*μσ*)
3	116.67482	3.889161±5.57115171
5	94.85629	3.161876±1.8519629
8	167.90354	5.596785±2.89910744
12	374.89234	12.49641±7.22735238
16	346.45443	11.54848±5.95766123

**Table 3:** Total decryption and mean and standard deviation (*μσ*) times for proposed SK4OA.

File Size (KB)	Total Decryption Time (ms)	Mean and Standard Deviation (*μσ*)
3	109.78892	3.659631±5.50655733
5	95.28016	3.176005±1.84544873
8	164.83854	5.494618±2.31357713
12	392.96387	13.0988±3.95820553
16	370.24271	12.34142±5.68254727

### 4.2 Simulation results for the proposed SK4OA

**Fig 3:**
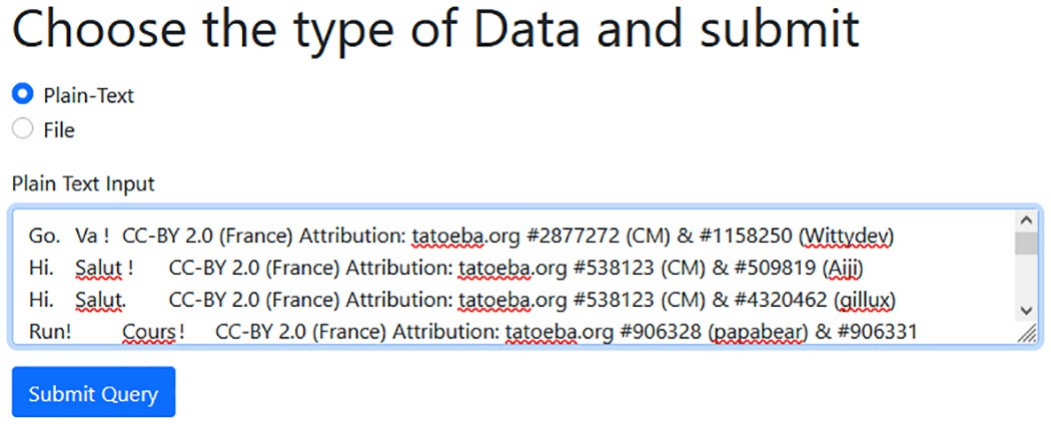
Plaintext of file size 2KB to be encrypted.

**Fig 4:**
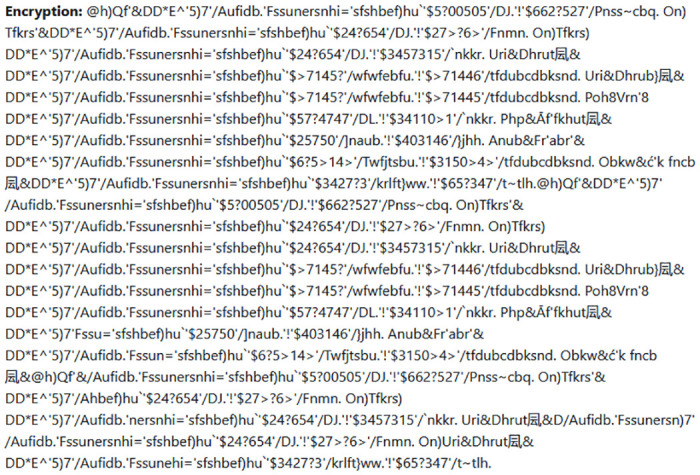
Encrypted results for 2KB data.

**Fig 5:**
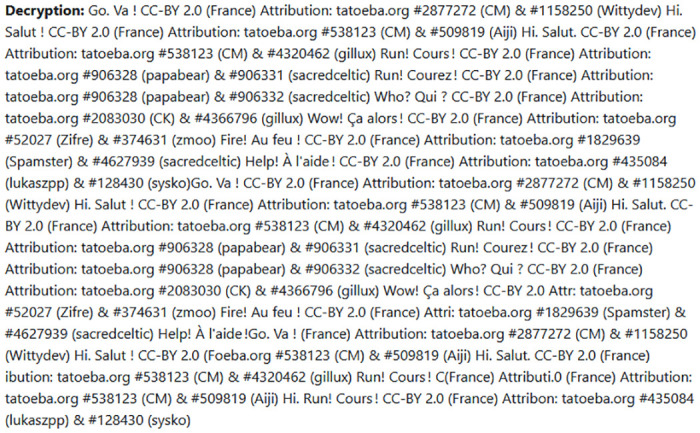
Decrypted results for a 2KB data.

This section presents the experimental graphical view of the required data needed to validate the proposed SK4OA cryptographic scheme. Fig 3 shows the plaintext, whereas Fig 4 shows the resultant Ciphertext. The plaintext is transformed to CHAR, and the decoded text is presented in Fig 5.

A total encryption time of 116.67482 milliseconds was derived from Table 2 with a data size of 3KB with a corresponding mean and standard deviation (*μσ*) times of 3.889161±5.57115171 milliseconds. With a comparable (*μσ*) time of 3.161876±1.8519629 milliseconds, the encryption time fell to a total encryption time of 94.85629 milliseconds with a data size of 8KB. The total encryption time rose to 167.90354 milliseconds when the data size was raised to 8 KB, however. With a data size of 12 KB, the total encryption time increased once more to 374.89234 milliseconds, with an equivalent (μσ) of 12.49641±7.22735238 milliseconds. The total encryption time decreased to 346.45443 milliseconds with a (*μσ*) time of 11.54848±5.95766123 milliseconds while the data size was increased to 16 KB.

From Table 3, a total decryption time of 109.78892 milliseconds was recorded for the proposed SK4OA algorithm with a data size of 3KB and a corresponding *μσ* time of 3.659631±5.50655733 milliseconds. The total decryption time decreased to 95.28016 milliseconds but increased to 164.83854 and 392.96387 milliseconds when data sizes were increased from 8KB to 16 KB respectively. However, the total decryption time decreased again to 370.24271 milliseconds with a *μσ* time of 12.34142±5.68254727 milliseconds when the data size was increased to 16 KB.

## 5. Discussion

### 5.1 Comparison of proposed SK4OA with RC4, SALSA20, and CHACHA20

To ascertain the algorithm’s performance, a comparison was made with state-of-the-art stream cipher algorithms such as RC4, Salsa20, and Chaca20. Tables 4 and 5 compares the encryption and decryption times of RC4, Salsa20 and Chacha20.

**Table 4:** Comparing the mean and standard deviation (*μσ*) encryption time of the proposed SK4OA algorithm against RC4, Salsa20, Chacha20 utilizing different data sizes.

Algorithm	*μσ* Encryption Time (Milliseconds)				
	3KB	5KB	8KB	12KB	16KB
RC4	**1.556±0.522**	3.462±1.184	6.297±1.551	12.364±2.602	16.215±4.103
Salsa20	2.589±0.412	3.822±0.374	5.790±0.994	7.617±0.941	14.282±1.190
Chacha20	1.694±0.258	2.959±0.427	4.698±0.954	**6.664±1.035**	11.004±1.220
SK4OA	2.552±3.539	**1.933±0.956**	**1.644±2.028**	7.214±1.952	**5.545±2.785**

**Table 5:** Comparing the mean and standard deviation (*μσ*) decryption time for the proposed SK4OA algorithm against RC4, Salsa20, Chacha20 algorithms utilizing different data sizes.

Algorithm	*μσ* Decryption Time (Milliseconds)				
	3KB	5KB	8KB	12KB	16KB
RC4	**1.493±0.363**	3.109±1.429	5.692±1.666	12.132±3.600	18.075±3.009
Salsa20	3.732±0.934	4.868±0.714	6.484±0.969	8.011±0.915	15.408±1.1555
Chacha20	1.654±0.265	3.167±0.719	5.244±1.164	**7.641±1.317**	12.220±1.473
SK4OA	2.099±2.523	**1.974±0.910**	**1.881±1.451**	7.944±2.1945	**5.149±2.231**

Table 4 shows that RC4 had an *μσ* encryption time of 1.556±0.522 milliseconds and a data size of 3KB. When the data size was extended to 5KB, 8KB, 12KB, and 16KB, the *μσ* run times climbed to 3.462±1.184 milliseconds, 6.297±1.551 milliseconds, 12.364±2.602 milliseconds, and 16.215±4.103 milliseconds, respectively, making their run times dependent on data size (O (N)). Table 4’s Salsa20 and Chacha20 likewise showed growing run times as data sizes rose (O (N). The suggested scheme SK4OA with a data size of 3 KB had a *μσ* encryption time of 2.552±3.539 milliseconds. With a data size of 5KB, this time was reduced to 1.933±0.956 milliseconds again for the proposed SK4OA algorithm. Also, the *μσ* encryption time decreased to 1.644±2.028 milliseconds for data size of 8KB. However, the *μσ* encryption time increased to 7.214±1.952 milliseconds, and decreased to 5.545±2.785 milliseconds as data sizes increased from 8KB to 12KB and 16KB correspondingly.

From Table 5, with a data size of 3KB, Chacha20 had a *μσ* decryption time of 1.654±0.265 milliseconds. This increased to 3.167±0.719 milliseconds, 5.244±1.164 milliseconds, 7.641±1.317 milliseconds and 12.220±1.473 milliseconds for data sizes of 5KB, 8KB,12KB and 16KB respectively. The *μσ* decryption times for RC4 and Salsa20, from Table 5, are linear (*O*(*N*)) as run time is directly proportional to data size. The proposed algorithm SK4OA had a *μσ* decryption times of 2.099±2.523 milliseconds for a data size of 3KB. The *μσ* decryption times decreased to 1.974±0.910 milliseconds. It further decreased to 1.881±1.451 milliseconds and increased to 7.944±2.1945 milliseconds when data size increased to 12KB. However, the *μσ* decryption times decreased again to 5.149±2.231 milliseconds when the data size was increased to 16KB.

From Tables 4 and 5, RC4, Salsa20 and Chacha20, had linear (O (N)) mean encryption and decryption times [37]. This implies that encryption and decryption times increase as data sizes increased [14, 30, and 38]. Tables 4 and 5, indicates that RC4, had the lowest encryption and decryption standard deviations of ±0.522 and ±0.363 for data size of 3KB. This implies that, the encryption and decryption times are clustered tightly around the mean, signifying patterned run times [39]. However, the proposed algorithm SK4OA had the highest encryption and decryption standard deviations of ±3.539 and ±2.523 for data sizes of 3KB. This indicates that the encryption and decryption times were more spread out, signifying non- deterministic run times [39–41].

#### 5.1.1 Throughput time

Any security algorithm’s throughput provides an indication of how quickly it performs encryption and decryption operations. The throughput time is computed using Eq 8.


$$Throughput\;Time=\frac{Data\;Size\;\left(KB\right)}{Run\;Time\;\left(ms\right)}$$ (8)

From Table 6, with a data size of 3KB, RC4 had the highest mean encryption throughput time of 1.928 KB/ms followed by Cacha20 (1.771 milliseconds). When the data size was increased to 5KB, the proposed algorithm had the highest mean encryption throughput time of 2.587 KB/ms. The encryption throughput for SK_4_OA increased to 4.866 KB/ms when the data size was increased to 8KB. However, when the data size was increased to 12KB, RC4 had the lowest encryption throughput time of 0.971KB/ms. However, with a data size of 16KB, SK4OA had the highest mean encryption throughput time of 2.885 KB/ms.

**Table 6:** Comparing the mean encryption throughput time of the proposed SK4OA scheme against RC4, Salsa20, Chacha20 algorithms utilizing different data sizes.

Algorithm	*μ* Encryption Throughput Time (KB/ms)				
	3KB	5KB	8KB	12KB	16KB
RC4	1.928	1.444	1.270	0.971	0.987
Salsa20	1.159	1.308	1.382	1.575	1.120
Chacha20	1.771	1.689	1.703	1.801	1.454
SK4OA	1.176	2.587	4.866	1.663	2.885

With a data size of 3 KB, RC4 had the highest mean decryption throughput time of 2.009 KB/ms from Table 7. However, with a data size of 16 KB, RC4 had the lowest mean decryption throughput time of 0.885 KB/ms. When the data size was increased to 5 KB, SK4OA had the highest mean decryption throughput time of 2.533 KB/ms.

**Table 7:** Comparing the mean decryption throughput time of the proposed SK4OA scheme against RC4, Salsa20 and Chacha20 algorithms utilizing different data sizes.

Algorithm	*μ* Decryption Throughput Time (Milliseconds)				
	3KB	5KB	8KB	12KB	16KB
RC4	2.009	1.608	1.405	0.989	0.885
Salsa20	0.804	1.027	1.234	1.498	1.038
Chacha20	1.814	1.579	1.526	1.570	1.309
SK4OA	1.429	2.533	4.253	1.511	3.107

According to Tables 6 and 7, the *μ* encryption and decryption times are inversely proportional to throughput time. There is lesser utilization of CPU when encryption and decryption times are higher and lower with higher throughput time [42].

#### 5.1.2 Memory usage

From Tables 8 and 9, Chacha20 had the lowest *μσ* encryption and decryption times for data sizes of 3KB, 5KB, 8KB, and 12KB. However, the same *μσ* sizes of memory was utilized by SK4OA for data sizes of 8KB and 12KB.

**Table 8:** Comparing the amount of memory utilized by SK4OA and other state-of-the-art algorithms (RC4, Salsa20, Chacha20) during encryption.

Algorithm	*μσ* Encryption Memory Size (MB)				
	3KB	5KB	8KB	12KB	16KB
RC4	3.832±0.890	5.131±0.942	7.395±1.369	8.977±1.892	15.541±0.928
Salsa20	3.732±0.934	4.864±0.714	6.484±0.969	8.011±0.915	15.408±1.156
Chacha20	1.654±0.265	3.167±0.719	5.244±1.164	7.641±1.317	12.219±1.473
SK4OA	4.037±0.111	6.057±0.122	11.053 ±0.168	11.053 ±0.146	13.067 ±0.201

**Table 9:** Comparing the amount of memory utilized by SK4OA and other state-of-the-art algorithms (RC4, Salsa20, Chacha20) during decryption.

Algorithm	*μσ* Decryption Memory Size (MB)				
	3KB	5KB	8KB	12KB	16KB
RC4	3.832±0.890	5.131±0.942	7.395±1.369	8.977±1.892	15.541±0.928
Salsa20	3.732±0.934	4.864±0.714	6.484±0.969	8.011±0.915	15.408±1.156
Chacha20	1.654±0.265	3.167±0.719	5.244±1.164	7.641±1.317	12.219±1.473
SK4OA	4.037±0.111	6.057±0.122	11.053 ±0.168	11.053 ±0.146	13.067 ±0.201

From Tables 8 and 9, it could be deduced that for RC4, Salsa20, and Chacha20, the *μσ* memory utilization increased as data sizes increased. However, the *μσ* memory utilization for the proposed SK4OA algorithm had similar *μσ* memory utilization for data sizes of 8KB and 12KB. Also, with a *σ* encryption and decryption memory of ±0.111, ±0.122, ±0.168, ±0.146, ±0.201, ±0.111, ±0.122, ±0.146, ±0.168 and ±0.201 respectively for data size of 3KB, 5KB, 8KB, 12KB and 16KB, the memory sizes are clustered around the mean [43]. This implies that, the size of the memory consumed is close to the mean and the difference of utilized memory is not very significant [44].

It could be deduced that the proposed algorithm SK4OA produced a lower, non-patterned, unpredictable and secret-key dependent run-times overcoming the theory of RC4, Salsa20 and Chacha20 as the fastest stream cipher algorithms. This again defeats the notion that Chacha20 uses less memory during execution [45]. From the experimental results, SK_4_OA could be considered as a lightweight algorithm that could be better employed in the cloud to attain high security as it uses less loops and has lower run times [46]. Also, SK4OA could be used in environment, where less memory is needed such as ubiquitous computing.

## 6. Conclusion

Cloud computing services are one of the most current innovations in information technology, and they provide several advantages to clients. Cloud information security is a major concern for any firm considering a cloud migration. Cryptographic systems are among the most secure methods of preventing unwanted access. However, these cryptographic algorithms have linear run times and are predictable. SK4OA, a cryptographic system with non-linear run times for cloud data security, is proposed in this paper. The proposed algorithm was run thirty (30) times with a dataset from the Kaggle database, and the mean and standard deviations were computed. The suggested scheme SK_4_OA was compared against state-of-the-art algorithms (RC4, Salsa20, and Chacha20) in terms of encryption time, decryption time, throughput, and memory utilization. When a data size of 3KB was executed thirty (30) times, RC4 had the shortest mean encryption and decryption times of 1.556±0.522 ms and 1.493±0.363 ms, respectively. Nevertheless, for data sizes of 5KB, 8KB, 12KB, and 16 KB, Secret Key_4_ Optimization Algorithm (SK4OA) had the shortest mean encryption and decryption times. Again, SK4OA generated non-patterned execution times, making it resistant to hacking since hackers cannot forecast the run times and instead hack depending on the amounts of the data to be executed. According to the results of the investigation, the suggested scheme had the highest throughput time, showing reduced CPU engagement during execution. Again in comparing memory consumption for RC4, Salsa20, and Chacha20, memory utilization rises as data sizes rise in those schemes. However, the memory utilization difference for SK4OA for all the data sizes especially 8KB and 12 KB is insignificant, indicating the economy of memory usage by the proposed SK4OA. Future studies should be conducted in evaluating the performance of the proposed algorithm on higher systems considering performance parameters such as encryption time, decryption time, throughput time, and memory usage.
